# A simulation model approach to analysis of the business case for eliminating health care disparities

**DOI:** 10.1186/1471-2288-11-31

**Published:** 2011-03-19

**Authors:** David R Nerenz, Yung-wen Liu, Keoki L Williams, Kaan Tunceli, Huiwen Zeng

**Affiliations:** 1 Center for Health Services Research, Henry Ford Health System, Detroit, MI, USA; 2 Department of Industrial and Manufacturing Systems Engineering, University of Michigan-Dearborn, USA; 3 Deparatment of Economics, Wayne State University, Detroit, MI, USA

## Abstract

**Background:**

Purchasers can play an important role in eliminating racial and ethnic disparities in health care. A need exists to develop a compelling "business case" from the employer perspective to put, and keep, the issue of racial/ethnic disparities in health care on the quality improvement agenda for health plans and providers.

**Methods:**

To illustrate a method for calculating an employer business case for disparity reduction and to compare the business case in two clinical areas, we conducted analyses of the direct (medical care costs paid by employers) and indirect (absenteeism, productivity) effects of eliminating known racial/ethnic disparities in mammography screening and appropriate medication use for patients with asthma. We used Markov simulation models to estimate the consequences, for defined populations of African-American employees or health plan members, of a 10% increase in HEDIS mammography rates or a 10% increase in appropriate medication use among either adults or children/adolescents with asthma.

**Results:**

The savings per employed African-American woman aged 50-65 associated with a 10% increase in HEDIS mammography rate, from direct medical expenses and indirect costs (absenteeism, productivity) combined, was $50. The findings for asthma were more favorable from an employer point of view at approximately $1,660 per person if raising medication adherence rates in African-American employees or dependents by 10%.

**Conclusions:**

For the employer business case, both clinical scenarios modeled showed positive results. There is a greater potential financial gain related to eliminating a disparity in asthma medications than there is for eliminating a disparity in mammography rates.

## Background

Disparities in health among racial and ethnic groups in the US are well documented [1]. Although important exceptions can be found, a general pattern of poorer health (e.g., life expectancy, self-reported health status, incidence of disease) exists among members of racial or ethnic minority groups. One contributing factor to disparities in health is disparities in quality of health care; a similarly extensive literature documents disparities among racial and ethnic groups in terms of access to care and quality of care received [2,3]. Disparities in quality refer to differences in levels of quality (based on defined measurable indicators) that have no clinical justification for members of different racial/ethnic groups [4].

Some disparities in health and health care reflect the combined effects of poverty and lack of health insurance [5]. Evidence of disparities in quality of care exists among employed and insured individuals, even among individuals in a single type of insurance or a single health plan [6-8]. Demonstration projects supported by the Commonwealth Fund and the Health Resources and Services Administration (HRSA) have shown that data on enrollees' race/ethnicity can be linked to health plan quality of care data to generate reports that identify disparities in quality of care at the individual health plan level [9-11].

Purchasers can play an important role in eliminating disparities by requiring health plans to analyze quality of care data separately by race/ethnicity and provide regular reports; and by using a variety of mechanisms inherent in the purchaser-supplier relationship (e.g., incentive payment systems) to focus attention on the disparity problem and change clinical practice [12,13]. Thus a need exists to develop a compelling "business case" from the employer perspective to put, and keep, the issue of racial/ethnic disparities in health care on the radar screen [14]. A "business case" from the employer perspective inevitably will be somewhat different from one created from the health plan or provider perspective. Reductions in direct health care expenditures that follow from improved quality of care may or may not be relevant, for example, depending on whether the employer or a health plan is the at-risk entity in any specific time period.

In principle, the several components to a "business case for disparity reduction" from the employer perspective include, but are not necessarily limited to:

• additional direct medical expenses attributable to disparities in quality of care;

• costs of absenteeism, or lack of productivity while at work (sometimes called "presenteeism") related to poor health status that follows from health care disparities;

• costs of absenteeism or diminished productivity related to receiving medical care for preventable conditions or complications;

• costs of absenteeism or diminished productivity related to employees' obligations as caregivers for children or other family members (e.g., mother or father has to take day off from work when child with asthma has an exacerbation);

• costs of training or hiring new staff when employees are disabled, retired, or die;

• costs of specific interventions or general system changes designed to reduce or eliminate disparities;

• potential additional costs of medical care as a result of eliminating disparities (e.g., additional costs of antidepressant medications among minority patients who were previously under-treated).

The elements of a business case for disparity reduction are similar to, perhaps even identical to, the elements of a business case for quality improvement. In both domains, the activities in question include data collection and analysis, identification of processes or outcomes in which quality is less than an ideal or target level, interventions of various types to improve quality, and additional cycles of data collection and analysis to determine whether quality has improved. Most of this work lies outside the usual routine of medical care for which payment is provided, so additional costs are incurred for which some sort of business case is required.

We illustrate both a method for calculating an employer business case for disparity reduction and a comparison of the business case in two clinical areas. We conducted analyses of the direct (medical care costs paid by employers) and indirect (absenteeism, productivity) effects of eliminating known racial/ethnic disparities in mammography screening and appropriate medication use for patients with asthma. We chose these two clinical domains because they represent areas in which disparities in specific quality of care measures have been documented and because a body of published data exist linking disparities in quality of care to medical care costs and data on absenteeism and productivity. We aimed to determine whether a positive business case exists for disparity reduction in either clinical case scenario in an effort to emphasize the importance for employers' ongoing participation in disparity reduction efforts.

### Clinical Settings for Employer Business Case Modeling

#### Breast Cancer

Breast cancer is the second leading cause of cancer death among women. Each year, approximately 200,000 women in the US are diagnosed with breast cancer, and 40,000 will die of the disease [15]. Treatment can involve a difficult regimen of surgery, radiation therapy, chemotherapy, and reconstructive surgery for those women undergoing mastectomy. Even women who experience five-year disease-free survival have reduced quality of life during relatively extensive periods of treatment [16].

The probability of cure or extended remission of breast cancer depends on the stage of disease at diagnosis [17]. Mammography is part of a recommended set of breast cancer screening procedures (along with clinical breast exam and breast self-examination) whose main objective is diagnosis at the earliest possible stage of disease. Some variability exists among national guidelines for mammography frequency, but most guidelines recommend annual mammography for women over 50 [18]. The main benefit of increased mammography rates is in shifting the distribution of stage at diagnosis, and therefore the subsequent survival probabilities, among those women diagnosed with breast cancer [19]. The National Committee for Quality Assurance (NCQA) Health Employer Data and Information Set (HEDIS) for managed care plans includes a measure of mammography use, which is defined as the percentage of women between 50 and 65 who have been continuously enrolled in the plan for two years and have had at least one mammogram in the past two years [20].

There is some controversy about the extent to which there are meaningful racial/ethnic disparities in mammography rates. Recent national surveys have suggested that black, Hispanic, and white women all receive mammograms at approximately the same rate [21]. However, analyses of Medicare and other claims data sets typically show a disparity of 7-15 percentage points in mammography rates, with black and Hispanic women less likely to receive mammograms than non-Hispanic white women [22,23]. These disparities have been found within some individual managed care plans, suggesting that at least some number of plans will find disparities in mammography rates when analyzing their own HEDIS data [24].

Eliminating a disparity in mammography rates would involve raising rates in black or Hispanic women to the level currently found for white women. For a typical health plan with relatively high current mammography screening rates, that might involve increasing the mammography rate for minority women from 70% to 80%. The question we addressed in this simulation was: What would be the benefits to an employer in terms of attendance and productivity at work, and medical care costs, for an employed population of African-American women if a typical disparity of 10% in mammography rates were eliminated?

Mandelblatt et al [25] used a simulation modeling approach to estimate the costs and benefits of improving breast cancer outcomes among African-American women through either improved screening or improved treatments after diagnosis. Their results showed a relatively modest benefit for improved screening in a hypothetical cohort of 40-year-old African-American women. We used a similar modeling approach, but the analyses were focused on the 50- to 65-year-old age group that is the defined denominator population for the HEDIS breast cancer screening measures, and the annual mammography patterns being modeled were selected to match those typically found among members of managed care plans. We used published data specific to African-American women on mammography rates, distribution of stage at diagnosis, survival by stage, and days worked to estimate the benefits of eliminating disparities in mammography rates between African-American and white women.

#### Asthma

Asthma is a common chronic disease affecting 10-20 million Americans in 2003 [26,27]. (Variation in estimates depends on whether the specific measure is ever having had asthma, having had an asthma attack in the past 12 months, or report of currently having asthma.) Asthma is more prevalent in African-Americans and Hispanics than in non-Hispanic whites, so the burden of morbidity and mortality related to asthma occurs disproportionately in those groups [28]. Deaths from asthma are rare [29], but morbidity in the form of acute exacerbations and related days lost from work or school is common [30]. Data from national surveys and other studies suggest that members of minority groups experience more asthma-related lost work and school days than whites, even among those with comparable asthma severity levels [31,32].

National guidelines since 1991 recommend use of inhaled preventive medications, including inhaled corticosteroids (ICS), as the preferred method of long-term management for patients with persistent asthma [33]. (Patients with only mild, intermittent asthma may be appropriately managed with acute, "rescue" medications as needed.) Based on these guidelines, NCQA has included a measure of Appropriate Medications for Asthma in its HEDIS measures since 2000 [34]. The measure is defined as the proportion of health plan members with persistent asthma who have been prescribed at least one of the approved preventive medications in the past year. The most recent average rate reported by the NCQA for commercial managed care plans publicly reporting their HEDIS data was approximately 70% in 2008, and the 90^th ^percentile rate for such plans was approximately 80% [35].

Data from a number of studies suggest that African-American children and adults are less likely to receive prescriptions for preventive medications (and use them) than their white counterparts [36,37]. Since the medications are effective in all racial/ethnic groups and the national guidelines do not make different recommendations for different groups, disparities in use of recommended medications are presumed to be one of the key underlying reasons for disparities in morbidity and mortality [38,39]. Improving rates of appropriate medication use in minority patients should result in a reduction in disparities in measures like lost work or school days, emergency room (ER) visit rates, hospital admission rates, or deaths [40].

Our goal in this project was to model the effects of improvements in appropriate medication rates for children, adolescents, and adults, on lost school or work days and on direct medical care costs [41,42]. To link data on disparities in children's use of asthma medications to an employer business case, we made the assumption that an employed adult would have to take one day off work for every day in which a child had an asthma exacerbation requiring a physician office visit, an ER visit, or a hospital admission [43]. The model specifically focused on the effects of reducing or eliminating disparities in the HEDIS Use of Appropriate Medication for People with Asthma measure [44]. The modeling exercise asked the question of how many lost school or work days would be averted by increasing medication use rates in minority populations to those observed in non-Hispanic white populations. These benefits were estimated over time periods ranging from one to five years.

## Methods

In calculating a business case for reduction of disparities in HEDIS measures, we used Markov simulation models to estimate the consequences, for defined populations of African-American employees or health plan members, of a 10% increase in HEDIS mammography rates or a 10% increase in appropriate medication use among either adults or children/adolescents with asthma. These improvements would correspond to elimination of disparities of the magnitude often observed for those two measures. Although we also report findings in terms of quality-adjusted life years (QALYs) gained as a measure of benefit to patients and society of reducing disparities, the primary endpoints in modeling the employer perspective were direct medical care expenses and cost of days missed from work. Our main analytic goal was to determine whether there was a positive business case for disparity reduction in either area, and if so, to determine whether the case was stronger in one clinical area than another.

### Mammography Simulation Model

The basic approach used in this study was a Markov simulation model with annual cycles [45]. The model was set up to simulate the key events related to the diagnosis and treatment of breast cancer in a fixed cohort of African-American women aged 50-65, given different rates of mammography. All women in the simulation cohort started without cancer, and they either did or did not receive mammography in a given year. In each year, women may or may not have developed cancer; if cancer was diagnosed, the probability of advanced stage disease at diagnosis depended on whether mammography had been performed that year as part of an annual, biennial, or more infrequent schedule of mammography.

Women who did develop cancer in one year started the next year either in remission, with progressive disease, or dead, with probabilities depending on the stage at diagnosis during the year of diagnosis. Women who did not develop cancer in a given year started the next year without cancer and, again, either did or did not have mammograms. The model can be run for any desired number of years; for this study, the time period used was five years.

To assign values to the model, there were two health endpoints being modeled and two employment-related endpoints. In the mortality or survival analysis, the "dead" state was valued at 0 and all other states are valued at 1. For analysis of QALYs, each state was assigned a utility value between 0 and 1, based on values in published literature and a set of assumptions about how much of each year is spent in each state. (For example, a woman who dies of cancer in a given year does not die on the first day of the year, so she accumulates some QALY values during the part of the year in which she is alive.)

For analysis of employment endpoints, the probabilities of working and the average work loss days for those who are employed were estimated, on the basis of published literature, for each state in the model. Employment probabilities were estimated in an employment equation which included all the relevant factors affecting employment status - for example, breast cancer status, exogenous variables (age, race, education, etc.), and health insurance. Probit estimates were the most commonly used method to derive the probability of employment. The number of days lost from work due to undergoing treatment was also estimated. Then, monetary values of reductions in employment and increases in absenteeism for employers (e.g., cost to employer of replacing an employee missing a day of work due to illness) were calculated based on published data (see Table 1 for all model input parameters and sources).

**Table 1:** Input Parameters for Mammography Simulation Model

	Base Model Parameter	Range Used in Sensitivity Analysis	Source(s)
**State Transition Probabilities**			

Annual Incidence of Cancer	.003		[64-67]

Probability of Annual, Biennial, and Sporadic Mammogram Patterns	HEDIS 70% - Annual = 0.197 - Biennial = 0.395 - Sporadic = 0.270 - None = 0.138		[59-63], authors' calculations
	HEDIS 80% - Annual = 0.242 - Biennial = 0.483 - Sporadic = 0.182 - None = 0.093		

Increase in Probability of Mammography Following False Positive	.10 increase relative to probability in absence of false positive		[50]

Probability of Cancer Found, Given Screening Pattern	- Annual = .003 - Biennial = .003 - Sporadic = .005 - None = .008		[68,69]

Distribution of Stage at Diagnosis with (W/O) Mammogram	Annual - In Situ = 0.13 - Stage I = 0.41 - Stage II = 0.37 - Stage III = 0.07 - Stage IV = 0.02		[70-76]
	Biennial - In Situ = 0.13 (0.04) - Stage I = 0.41 (0.32) - Stage II = 0.37 (0.43) - Stage III = 0.07 (0.13) - Stage IV = 0.02 (0.08)	In Situ = 0.04 - 0.21 Stage I = 0.32 - 0.57 Stage II = 0.16 - 0.43 Stage III = 0.04 - 0.13 Stage IV = 0.02 - 0.08	
	Sporadic - In Situ = 0.17 (0.04) - Stage I = 0.29 (0.32) - Stage II = 0.40 (0.43) - Stage III = 0.09 (0.13) - Stage IV = 0.05 (0.08)		
	None - In Situ = (0.04) - Stage I = (0.32) - Stage II = (0.43) - Stage III = (0.13) - Stage IV = (0.08)		

Probability of Remission in Year of Diagnosis, Given Stage at Diagnosis	Stage I = 97.5% Stage II = 94.0% Stage III = 81.0% Stage IV = 66.0%		[73,77]

Probability of Death from Other Causes	.01		[78,79]

Probability of Continued Remission vs. Recurrent Disease, Given Stage at Diagnosis and Initial Remission	Stage I = 0.975 Stage II = 0.94 Stage III = 0.81 Stage IV = 0.66		[73,77]

Probability of Death, Given Recurrent or Progressive Disease	0.79		[73,77]

**State Values**			

Utility of Well, Non-Cancer States	1.0		[80-86]

Utility of Newly Diagnosed Cancer, by Stage at Diagnosis	Stage I = 0.90 Stage II = 0.85 Stage III = 0.81 Stage IV = 0.81	Stage I = 0.80 - 0.98 Stage II = 0.80 - 0.98 Stage III = 0.80 - 0.98 Stage IV = 0.80 - 0.98	[80-86]

Utility of Undergoing Treatment, by Stage at Diagnosis	Stage I = -0.10 Stage II = -0.20 Stage III = -0.25 Stage IV = -0.25		[80-86]

Utility of Progressive Disease	0.40	0.30 - 0.69	[80-86]

Probability of Employment - no Cancer	1.00		Arbitrary - simulation assumes women employed

Probability of Employment in Year of Cancer Diagnosis, by Treatment Stage	In Situ = 0.86 Stage I = 0.70 Stage II = 0.60 Stage III = 0.50 Stage IV = 0.40		[88-94]

Probability of Employment in Year of Cancer Diagnosis, by Remission Stage	Stage I = 0.94 Stage II = 0.88 Stage III = 0.81 Stage IV = 0.75		[88-94]

Probability of Employment - Progressive Disease	0.56		[88-94]

**Other Values**			

Costs of Initial Treatment, by Stage at Diagnosis	Stage I = $ 22,488.00 Stage II = $ 27,213.00 Stage III = $ 29,220.00 Stage IV = $ 31,476.00		[48]

Costs of Treatment - Recurrent or Progressive Disease	$ 33,000.00		[48]

Cost of Mammogram	$ 81.86		Federal Register, 2007

Cost of Followup after False Postive Test	$533		[106]

Hourly Wage for Time Missed from Work	$ 21.31		Department of Labor, May 2007 [46]

In general, we used estimates of absenteeism among women remaining employed, rates of women leaving work entirely, and medical care costs to employers for the six months following a new breast cancer diagnosis. All three are costs to employers that could be affected by increasing mammography rates and consequent shifts to earlier stage diagnoses. Based on the annual average wage of $21.31 per hour [46], the cost of lost productivity to the employer is the hours lost multiplied by hourly wage, assuming 8 hours worked per day and that absenteeism is the only cause of lost productivity.

When employees decide or must leave the workforce because of the severe illness, a turnover cost is incurred. The turnover cost to the employer comprises the expenses of recruiting and training new employees. Turnover costs generally include time and monetary costs to select, recruit and train a replacement, and lost productivity. A study based on the US Department of Labor estimated the costs to replace an employee to average 33 percent of the new hire's salary [47]. Assuming 2080 hours per year, using the estimated average hourly wage, the annual turnover cost is $14,627.

Medical costs of employed patients are also a burden for employers. In the model, costs for breast cancer care paid by private insurers (as estimated from available published literature) were presumed to represent costs to employers, since this would be literally true for self-insured employers, and true in a less direct sense for other employers as their insurance premiums would reflect recent costs of employees' cancer care. Initial and terminal phases of breast cancer treatments are the most costly [48].

**Figure 1:**
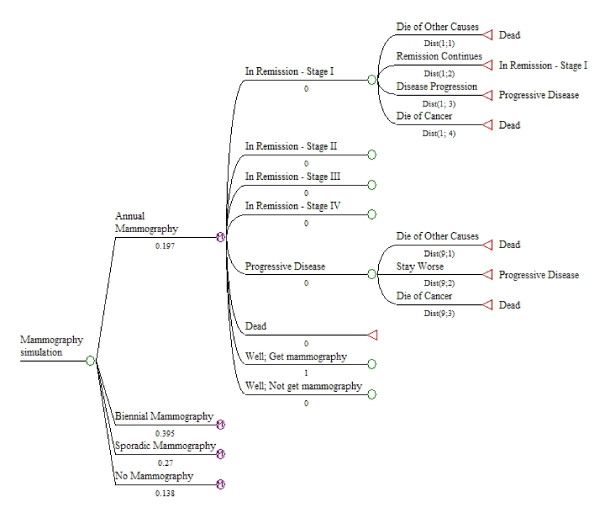
**Mammography pattern groups and starting states of simulation model**.

**Figure 2:**
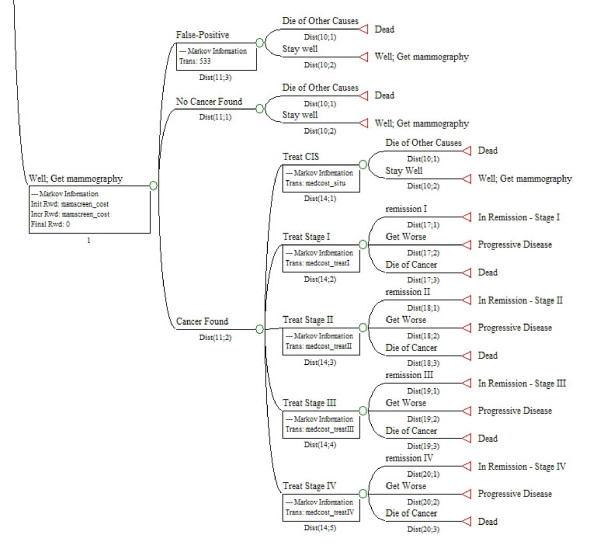
**Breast cancer stages and treatments in simulation models**.

Whenever possible, probabilities of events or utility values were obtained from studies of African-American women. The structure of the model is illustrated in Figures 1 and 2. The model was run for five annual cycles. All women started alive without cancer diagnosis in one of the four groups based on pattern of mammography, since the HEDIS denominator population definition excludes women with cancer diagnoses. Figure 1 shows the starting states for the model. At the beginning of the simulation, each woman in the simulation cohort was assigned to one of four groups based on her pattern of receipt of mammograms over multiple years - annual, biennial, sporadic, or none. The probabilities shown for each group reflect the probabilities of being in each group at the start of the simulation, for the 70% HEDIS rate scenario. Figure 1 also shows the set of states following each of the four "mammography pattern" groups.

Figure 2 shows the initial options for women who received a mammogram in a given year under any of the three patterns (annual, biennial, sporadic) that would include the receipt of a mammogram during a five-year simulation period. (The same options were included in the model for women not receiving mammograms, but are not shown here.) The key feature of the model is the stage of cancer at diagnosis, which determines the subsequent probabilities of remission and survival. The triangle symbols at the right side of the figure signify that women in those branches return to the specific named states to start the next annual cycle of the model, or die and no longer continue in the simulation cycle.

The input parameters for the model are summarized in Table 1. The key parameters were: probability of annual, biennial, and less frequent mammography; the incidence of breast cancer in the population of employed African American women aged 50-65; distribution of stage at diagnosis given a particular pattern of mammography; probability of remission or progressive disease following initial treatment given stage at diagnosis; probability of continued remission vs. recurrence given remission after initial treatment; probability of death given recurrence or progressive disease; annual probability of death from other causes in African-American women aged 50-65; utilities assigned to various disease, treatment, or disease-free states; probability of employment given cancer diagnosed at specific stages; and medical care costs associated with cancer diagnosed at specific stages.

#### Sensitivity Analysis

Sensitivity analyses were conducted to determine how the life expectancy, QALYs, employment rates, working hours missed plus turnover costs and medical care costs change when the distribution of stage at diagnosis in the simulation model moves from the most "pessimistic" one (i.e., least benefit due to mammography) to the most "optimistic" one (i.e., greatest effect of mammography on stage). In addition to the base model described, four more versions of the model with different combinations of distribution of cancer stage from Table 1 were defined and used for sensitivity analysis. Based on the different combinations, models are considered as moving from the most pessimistic to the most optimistic models. The definitions of all five models are summarized in the Appendix.

The base model did not include the costs or effects of false-positive mammograms, but one recent review suggested that over 10% of mammograms will be read as suspicious for cancer and then followed up with biopsy or other tests, even though cancers are not found in the vast majority of these [49]. Two additional versions of the model were created - one with an explicit cost added for false positive tests requiring additional followup, and another with those costs included and a slight increase in the subsequent probability of mammography after a false positive test [50].

Other input parameters were not included in sensitivity analyses either because they had one widely accepted value (e.g., annual cancer incidence rate) or because they could be set arbitrarily to fit any specific new application of the model (e.g., increase in mammography rate; hourly rate for employees). The relationship between mammography and stage distribution at diagnosis seemed to be the relationship with greatest variation in published values and greatest potential impact on model outputs.

##### Asthma Basic Model Structure

**Figure 3:**
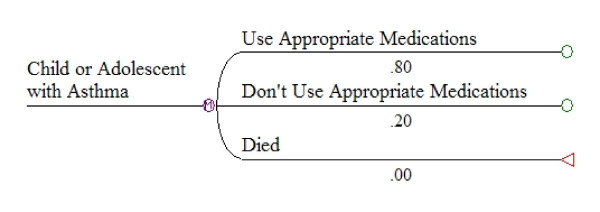
**Initial states for Markov model of appropriate medications for asthma**.

The basic modeling approach was a Markov model simulating the likelihood of various events like acute exacerbations, ER visits or hospital admissions, lost school or work days, or deaths as a function of taking or not taking appropriate medications. The model (illustrated in Figure 3) was structured to capture the range of possible events during a month. Hypothetical patients in the model began each month either taking or not taking appropriate medications, and then experienced events during the month with probabilities that depended on whether or not they were taking medications. At the end of each month, patients "cycle back" and began a new month either taking or not taking appropriate medications. The model was run for 12, 24, 36, 48, or 60 monthly cycles to simulate experience over time periods from one to five years.

The model is similar in structure to that developed by Paltiel et al [51], but does not include forced expiratory volume (FEV1) as a core concept, because we were unable to find adequate data for that approach specific to individual racial/ethnic minority groups. When possible, parameters from the Paltiel model were used in our model to try to maximize comparability across the two modeling approaches.

**Figure 4:**
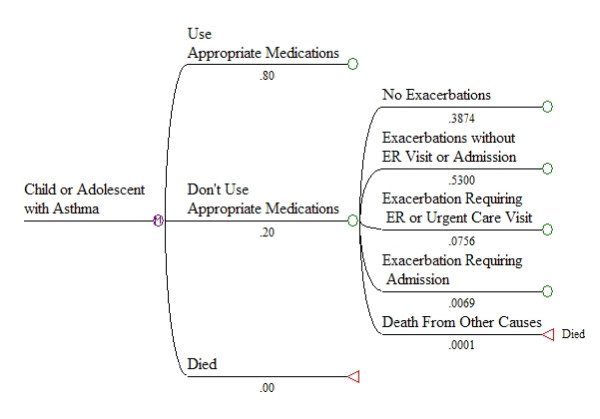
**Second set of state transition options, illustrated for the initial state of not using appropriate medications**.

**Figure 5:**
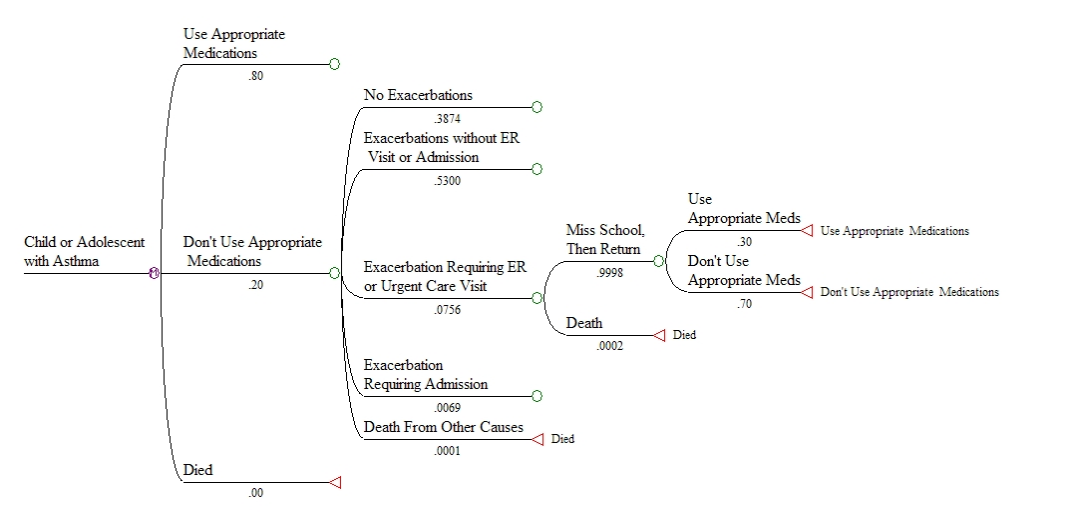
**Additional state transition options, given the occurrence of an exacerbation requiring an ER or urgent care visit**.

The structure of the model is illustrated in Figures 3, 4, and 5. Figure 3 shows the first set of branches, or starting states. Figure 4 shows the next set of state transition options, with associated monthly probabilities in the situation where a child is not using appropriate medications. The same transition options exist in the model for children using appropriate medications, but with different probabilities. Figure 5 illustrates the complete set of transition states, for a child not using appropriate medications and experiencing an exacerbation requiring an ER or urgent care visit during the month. Similar sets of branches follow from other "exacerbation" branches, but with different probabilities.

The primary input parameters for the model are summarized in Table 2. They included: probability of use of appropriate controller medications in a month, probability of exacerbations in a month given use or non-use of controller medications, probability of ER or urgent care visit or hospitalization given exacerbation, number of days missed from work given exacerbations of varying severity, medical care costs associated with treatment of exacerbations of varying severity, costs of controller medication [52], and utilities associated with specific states of exacerbations and freedom from exacerbations. The models were run for both adults and children; for children, days missed from school was used instead of days missed from work, but in analyzing days missed from work to calculate a business case from the employer perspective, it was assumed that an employed parent would miss a day of work for every day a child with asthma was out of school because of an exacerbation.

**Table 2:** Input Parameters for Asthma Medication Model

	Base Model Parameter	Range Used in Sensitivity Analysis	Source(s)
**State Transition Probabilities**			

Probability of Asthma Medication Use	80%	70% (90%)	[37,95,96,99]

Probability of Exacerbation not requiring ER visit or Admission	With Medication = 0.27 W/O Medication = 0.53	Same as 80%	[32,95,98]

Probability of Exacerbation Requiring ER Visit, not Admission	With Medication = 0.0382 W/O Medication = 0.0756	Same as 80%	[95,98,102-104]

Probability of Exacerbation Requiring Admission	With Medication = 0.0035 W/O Medication = 0.0069	Same as 80%	[49,101]

Probability of Medication use Following Exacerbation	With Medication - no ER visit or Admission = 0.96 - ER visit, no Admission = 0.99 - Admission = 0.99	With Medication - no ER visit or Admission = 0.97 (0.99) - ER visit, no Admission = 0.99 (0.99) - Admission = 0.99 (0.99)	Authors' values, constrained by requirement that overall medication use be constant over entire simulation
	W/O Medication - no ER visit or Admission = 0.25 - ER visit, no Admission = 0.30 - Admission = 0.30	W/O Medication - no ER visit or Admission = 0.15 (0.4) - ER visit, no Admission = 0.25 (0.45) - Admission = 0.25 (0.45)	

Probability of Medication Use Following Month of No Exacerbation	With Medication = 0.96 W/O Medication = 0.01	With Medication = 0.95 (0.965) W/O Medication = 0.01	Authors' values, constrained by requirement that overall medication use be constant over entire simulation

Probability of Death from Other Causes	0.0001	Same as 80%	

**State Values**			

Utility of Month without Exacerbation	With Medication = 0.0748 W/O Medication = 0.0756		[49]

Utility of Exacerbation not requiring ER visit or Admission	With Medication = 0.0706 W/O Medication = 0.0712		[49]

Utility of Exacerbation requiring ER visit, no Admission	With Medication = 0.0698 W/O Medication = 0.0704		[49]

Utility of Exacerbation requiring Admission	With Medication = 0.0649 W/O Medication = 0.0654		[49]

Days Missed per month from Work or School from Exacerbation not requiring ER visit or Admission	3 days		[95,98]

Days Missed from Work or School from Exacerbation requiring ER visit, no Admission	4 days		[95,98]

Days Missed from Work or School from Exacerbation requiring Admission	7 days		[33,98]

Cost of Doctor Office Visit for Exacerbation	$155.44		[105], authors' calculation

Cost of ER Visit	$1,080.00		[105], authors' calculation

Cost of Admission	$13,512.00		[105], authors' calculation

Cost of Monthly Asthma Medications	$112.00		[50]

Hourly Wage for Time Missed from Work	$21.31		Department of Labor, May 2007 [46]

Patients are not consistent from month to month or year to year in their use of medications. Although a rate of adherence may be stable over time for a large population, individuals can alternate between periods of use and non-use, and the Markov model structure allows for this factor to be included. We presumed that medication use would be generally stable from month to month, but that some individuals taking medication and experiencing no exacerbations in a given month would not use medications in the following month (in various versions of the model we used proportions of 5-10%). Even some individuals experiencing ER visits or hospitalizations may not use medications in the following month (perhaps believing them to be ineffective); we estimated this proportion as 1%, with 99% of those experiencing ER visits or hospitalizations continuing on medications.

Among those not using medications in a given month, we presumed that 1% of those not experiencing exacerbations would begin to use medications in the following month, that 15% of those experiencing an exacerbation would begin to use medications in the following month, and that 25% of those requiring an ER visit or hospitalization would begin using medications in the following month.

In the absence of published data on these transitions among minority group patients with asthma, some of the transition probabilities are somewhat arbitrary. They were adjusted to produce the effect of gradually rising use of medication for the group as a whole over time. This trend of gradual increase is consistent with national data on improvement in the HEDIS asthma medication measure over time [9].

## Results

### Breast Cancer

#### Life Expectancy

In Table 3, life expectancy is expressed as the life expectancy for a typical African-American woman in the HEDIS denominator population, over a five-year period, with mammography rates increasing by 10% from 70% to 80%. These results suggest that an increase of 10% in the mammography rate for African-American women would add a total of 15 years in a denominator population of 10,000 women, or 1-2 years in a denominator population of 1,000 women.

**Table 3:** Mammography Model Outputs, with Sensitivity Analysis Results

Model Output		Version of Simulation Model*				
	**Rate**	**Base**	**I**	**II**	**III**	**IV**

	70%	4.85705	4.85599	4.85731	4.85756	4.85772
	
Life Expectancy	80%	4.85856	4.85735	4.85887	4.85919	4.85929
	
	***Diff***	***0.00151***	***0.00136***	***0.00156***	***0.00163***	***0.00157***

	70%	4.84524	4.84369	4.84570	4.84617	4.84643
	
QALYs	80%	4.84762	4.84586	4.84820	4.84877	4.84894
	
	***Diff***	***0.00238***	***0.00217***	***0.00250***	***0.00260***	***0.00251***

	70%	4.84087	4.83922	4.84135	4.84183	4.84211
	
Employment Rate	80%	4.84357	4.84170	4.84417	4.84475	4.84494
	
	***Diff***	***0.00270***	***0.00248***	***0.00282***	***0.00292***	***0.00283***

	70%	$178.1132	$181.9811	$176.8969	$175.6906	$175.1368
	
Cost to Employer of Missed Work	80%	$165.5864	$169.9384	$164.0923	$162.6172	$162.2439
	
	***Diff***	***$-12.5268***	***$-12.0427***	***$-12.8046***	***$-13.0734***	***$-12.8929***

	70%	$1123.3621	$1142.1542	$1116.8214	$1110.3160	$1106.7010
	
Medical Care Cost	80%	$1085.6956	$1107.1215	$1077.6608	$1069.7061	$1067.2694
	
	***Diff***	***$-37.6665***	***$-35.0327***	***$-39.1606***	***$-40.6099***	***$-39.4316***

*Models vary in terms of the shift in distribution of stage at diagnosis attributed to increase in mammography rates. Model I is least optimistic in terms of the amount of stage shift produced: model IV is most optimistic.

#### Quality-Adjusted Life Years

Table 3 also shows the effect of a 10% increase in HEDIS mammography rate on the five-year cumulative QALYs for an African-American woman in the 50-65 age cohort. As in the case of life expectancy, the gain is relatively modest - approximately 2 QALYs gained per 1,000 women in the cohort over five years. Improving mammography rates for African-American women from 70% to 80% could be expected to add 24 quality-adjusted life years in a population of 10,000 women aged 50-64. The QALY gains are greater than the life expectancy gains because some of the women alive at the end of five years after having had cancer would have avoided the treatments associated with late-stage cancer at diagnosis by having more frequent mammography.

#### Costs Associated with Absenteeism and Workforce Turnover

In the scenario with a 70% HEDIS mammography rate among African-American women aged 50-65, an employer could expect to incur approximately $178 of cost per woman in the cohort related to absenteeism and employer turnover following breast cancer diagnosis and treatment in that cohort. When the mammography rate rises to 80% (disparity in mammography eliminated), the expected cost related to absenteeism and turnover dropped to approximately $166 (savings of $12 per woman in cohort) because of less absenteeism and turnover among women with less advanced stage of cancer at diagnosis. Therefore, in a cohort of 100 women, the projected savings would be $1,200 over five years; $12,000 in a cohort of 1,000 women, etc.

#### Direct Medical Care Costs

Similarly, an employer in the 70% HEDIS mammography scenario could expect to incur approximately $1,123 in medical care costs per employed African-American woman aged 50-65 related to new breast cancer diagnoses; that estimate became $1,086 as the HEDIS mammography rate rose to 80%. Again, the main reason for the effect is lower medical care cost associated with less advanced stage at diagnosis. The projected saving with a 10% increase in mammography rate is $38 per woman in an employed cohort over a five-year period.

#### The Employer "Business Case" - Combination of Absenteeism, Turnover, and Medical Care Costs

The combined savings per employed African-American woman aged 50-65 associated with a 10% increase in HEDIS mammography rate, from all three areas combined, was $50. For an employer with 100 African-American women in that age group, then, the projected savings associated with eliminating a 10% disparity in mammography rates would be $5,000. A quality improvement or health education initiative designed to enhance mammography screening, and that did in fact raise mammography rates by 10%, would have to cost $5,000 or less to be cost-saving.

#### Sensitivity Analysis

Several alternative versions of the model were run with varying assumptions about stage distributions with or without mammography. In the alternative models, 13-16 lives per 10,000 women would be saved as a result of increasing mammography rates in African-American women by 10%. The small effects of variations of the model reflect the low incidence of cancer in any one- or two-year period, and that the shift in stage at diagnosis as a result of mammography is relatively modest, even using the most optimistic data on stage distributions in the literature.

The results of these sensitivity analyses are shown in Table 3. The pattern observed across all major output parameters in the model is similar. As expected, a more optimistic estimate of the effect of mammography on stage distribution is associated with a larger effect on QALYs, cumulative employment, and costs to employers for time missed from work. The size of the differences across different versions of the model is small, suggesting that the model results are robust across all reasonable estimates of the effect of mammography on stage at diagnosis from the published literature.

Adding an explicit cost for false positive tests had a somewhat more significant effect on model outputs, although in all cases the direct medical care costs were still slightly lower in the 80% mammography scenario than in the 70% scenario. The cost saving was reduced from $50 to $18 in the model that included just the direct cost of false positives ($1,248 vs. $1,230), and to $17 in the model that included both costs of false positives and a slight increase in likelihood of mammography in subsequent cycles after a false positive.

### Asthma

#### Life Expectancy

Probabilities of death were extremely low in all versions of the model, and did not vary significantly with improvements in medication use rates from 80% to 90%.

#### Quality-Adjusted Life Years

The effect of increasing appropriate medication use on QALYs depends partly on assumptions made on the number of days spent at home during acute exacerbations, and the extent to which one applies a "QALY penalty" for spending time in an ER or hospital during an exacerbation. The effects of variations on these parameters are relatively small, though, so the results shown in Table 4 are typical of the range of QALY benefits to be obtained over five years by improving appropriate medication rates from 80% to 90% in African-American or Hispanic children or adults. There was a gain of approximately 1.5-2 QALYs per 1,000 people with asthma in the denominator population, per year, that would be obtained by eliminating a 10% disparity in appropriate medication use (raising the rate of medication use from 80% to 90%).

**Table 4:** Gains in QALYs, Work Days for Adults, and School Days for Children Associated with 10% Changes in Asthma Medication Use Rates

Model Output	Appropriate Medication Use Rate				Gain Per 10% Increase in Rate
	**60%**	**70%**	**80%**	**90%**	

**Cumulative QALYs - 5 Years**	4.37627	4.37996	4.38387	4.38738	3-4 QALYs Per 1,000 People

**Cumulative Work Days Over 5 Years - Adults**	1,216.39	1,221.74	1,227.41	1,232.49	5-6 Work Days Per Adult

**Cumulative School Days Over 5 Years - Children**	815.79	821.13	826.79	831.47	4-6 School DaYs Per Child

#### Costs Associated with Absenteeism

From the employer perspective, there is a gain of over one full work day per year per African-American employee with asthma by eliminating a disparity of 10% in use of appropriate medications. There was also a gain of over one school day per year per African-American child or adolescent with asthma by eliminating a disparity of 10% in use of appropriate medications.

A reduction in days missed from work of over one day per year, or five to six days over a five-year period, would yield a savings to the employer of $866.04 per adult employee with asthma over that five-year period. If we assume that an employed parent would take one day off work for every day missed away from school for a dependent child with asthma, the savings to that parent's employer associated with a reduction in disparity in medication use for children with asthma would be $852.40 per employed parent. The annual savings would total $343.69. The effects of more conservative assumptions about days missed by employed parents for children with asthma exacerbations are shown in Table 5.

**Table 5:** Comparison of Key Model Outputs Relevant to Employer Business Case for Disparity Reduction

Disparity-Reduction Intervention	Direct Medical Care Costs - Change Per Person	Days Off Work - Change Per Person	Indirect Costs - Change Per Person	Total - Change Per Person
Increase HEDIS Mammography Rate in African American Women by 10% (70%-80%)	$ - 37.66	-0.59	$ -12.53 (no turnover cost) $ -140.03	$ -50.19 (no turnover cost) $-177.69 (with turnover)
Increase HEDIS Appropriate Medication Use in Adults by 10% (80-90%)	$ - 793.72	- 5.08	$ -866.04	$ -1,659.76
Increase HEDIS Appropriate Medication Use in Adults by 20% (70-90%)	$ - 1,680.26	- 10.75	$ -1,832.66	$ -3,512.92

#### Direct Medical Care Costs

Eliminating a disparity of 10% in asthma controller medication use would produce a savings of $793.72 per patient in terms of reduction in costs associated with ER and urgent care visits and hospitalizations over a five-year period. However, when the additional controller medication costs associated with that 10% increase are included in the calculation, the overall savings in direct medical care costs associated with that 10% increase drop to $86.50 per person over the five-year period, or $17.30 per employee with asthma (or parent of child with asthma) per year.

### Comparison of Business Cases for Mammography vs. Asthma Medication

Table 5 summarizes the employer business case for reducing disparities either in mammography rates or in asthma medication use rates, using the total of direct medical care expenses and indirect expenses associated with days missed from work. Regardless of whether factoring into the calculation the turnover cost for women with breast cancer, there is a greater potential financial gain related to eliminating a disparity in asthma medications than there is for eliminating a disparity in mammography rates.

## Discussion

A number of caveats accompany the presentation of these findings. First, any simulation model inevitably involves some distortion of actual clinical events, although the model used here was designed to simulate the key events as closely as possible. Some potentially relevant and unaccounted-for clinical factors include disparities follow-up of abnormal mammograms, differences in access to treatments (beyond those already reflected in survival rates), or differences in subsequent mammography patterns as a result of positive or negative mammograms.

Second, the models and their calculations were inevitably dependent on the limitations of published data. For some parameters, available data were several years old, and may not fully reflect clinical practice today. Nevertheless, models can easily be updated to take into account new information as it becomes available.

Third, the model had to use some parameters that were not specific to African-American women with breast cancer or adults or children with asthma. Utilities for specific health states, for example, were taken from studies of large, multi-ethnic or predominantly white populations. Again, future models can take these factors into account as group-specific information becomes available.

Given these constraints, we were encouraged by the extent to which our findings matched those of other investigators using similar, but not identical, analytic approaches. Mandelblatt et al [25] estimated that two alternative approaches to improving mammography rates among African-American women would add approximately .0008 years of life expectancy to a 40-year-old woman over a period slightly in excess of 20 years. Our results are also reasonably consistent with a recent Canadian study showing 3 lives saved per 25,000 mammograms [53]. In all of these analyses, the health benefits of improved mammography rates are modest relative to other potential interventions like reducing disparities in cancer treatment or improving care for other clinical conditions [54]. Adopting a longer time perspective would increase these estimates, as women avoiding late-stage breast cancer because of more frequent mammography in any five-year period continue to survive for many years after this period. From a public health perspective, these gains in life expectancy over longer periods of time justify investments in mammography. A time horizon longer than five years is not typically used to judge the benefits of investments in quality improvement programs.

From the employer business case point of view, the effects of eliminating disparities in mammography on employer-related outcomes (cost of absenteeism, cost of employee turnover, and direct medical care expenses) were positive, but modest. A 10% increase in mammography rates is estimated to save $12-$13 per employed African-American woman over a five-year period due to reduced costs of absenteeism, and an additional $37-$38 per woman in reduced medical care expenses. There is a total savings to employers, then, of $50 per employed African-American woman over five years, following a 10% improvement in mammography rates, if one assumes no turnover ($17-18 if the costs are false positive mammograms are included); the savings is approximately $180 per employed woman if projected turnover costs are included.

Since we do not yet have a large literature on the costs of interventions needed to improve mammography rates among African-American women, it is not yet possible to extend the models into the realm of cost-effectiveness analysis and compare the cost-effectiveness of eliminating disparities in mammography rates to those of eliminating disparities in other areas like glycemic control in diabetes or medication adherence in asthma. The calculation above, though, suggests that a disparity-reduction intervention that can be carried out for less than $50-$180 per woman in the target group can be cost-saving.

The findings for asthma were more favorable from an employer point of view. The reduction in direct medical care expenses and the reduction in costs due to absenteeism or diminished productivity related to elimination of a disparity in medication usage were both significantly larger than those estimated for mammography. We also do not have data on costs of interventions to reduce disparities in asthma medication usage, but an intervention would be cost-saving if it could be carried out for less than $1,660 per person and if had the effect of raising medication adherence rates in African-American employees or dependents by 10%.

Similar analyses have been conducted to create a rationale for choosing measures for pay-for-performance programs or for prioritizing quality improvement initiatives [55-57]. As more data become available with which to populate simulation or decision analysis models, it should be possible to have an empirical basis for deciding among competing priorities for disparity-reduction initiatives, and for developing an employer business case for those initiatives. A strong employer business case would help support efforts by health plans and provider organizations to reduce and eliminate disparities [58].

The simulation model approach illustrated here could be used to address related questions in a broader set of disparities in domains of disease prevention, early detection, and disease treatment. In general, the models require one or more well-defined endpoints of either clinical or economic significance (e.g., deaths, QALYs, costs) and then information necessary to define the probabilities of essential intermediate clinical or health states leading to those endpoints. For example, a model of the business case for addressing disparities in treatment for early-stage lung cancer would include probabilities of surgery for early-stage cancer for various racial/ethnic groups, probability of recurrence or progression following surgery, probability of recurrence or progression following other treatment approaches (including no treatment), duration and quality of survival given alternative treatments, and patterns of employment and work attendance given alternative treatments and related disease trajectories. The specific form and key elements of various models will naturally vary from one to the other, but we hope that the examples described here will illustrate the potential of the approach.

## Conclusions

A simulation model approach can be used to estimate the benefits to employers and to other stakeholders of initiatives to reduce racial/ethnic disparities in quality of health care. The results of these simulation models can serve as part of a "business case" for disparities reduction. Using mammography screening and asthma medication use as examples, we demonstrate that this approach can be used to compare the benefits of disparity reductions in different clinical domains.

## Competing interests

The authors declare that they have no competing interests.

## Authors' contributions

DRN was responsible for overall study design, development of initial simulation models, selection of input parameters, and drafting and editing of manuscript. YL was responsible for enhancements to simulation models, sensitivity analyses and exploration of alternative model structures, and editing of manuscript. LKW was responsible for reviewing model structure and input parameters for asthma simulation model and for editing of manuscript. KT was responsible for model parameters on absenteeism, productivity, and costs to employers, for direct and indirect cost analysis, and for editing of manuscript. HZ was also responsible for model parameters on absenteeism, productivity, and costs to employers, and for editing of manuscript. All authors have read and approved the final manuscript.

## Appendix - Detail on Model Parameters

### I. Breast Cancer Screening Model

#### State Probabilities

##### Mammography

Reisch et al [24] reported a 6% difference in rates of screening mammography between African-American and white women enrolled in a health maintenance organization (HMO) from 1983 to 1990, and a 9% difference in the first part of that period (1983-1987). Corresponding national figures for the same time periods showed a disparity in mammography screening rates of 7-10%. Burns et al [59] reported a 6% difference in mammography rates between white and black women over 65, using Medicare Part B billing files from 10 states in 1990. O'Malley et al [60] reported a larger disparity among women in North Carolina in 1988, with 36% of white women reporting having had a mammogram in the past year vs. 17% of black women.

Data from the 1998 National Health Interview Survey showed a 4.7% disparity in "recent mammograms" among women over 65 [61]; a slightly smaller disparity (3.6%) was noted in the same data set between African-Americans and whites among women aged 40-64 [62]. Rates for Hispanic women aged 40-64 were 10.8% lower than those of white women.

Mammography rates have been rising since some of these data were collected in the 1980s, and data from the NCQA's State of Health Care Quality report in 2005 (when the age bands for the HEDIS mammography measure were the same as used in our analysis) show the mean national screening rate in HMOs at 72.0% for commercial enrollees and 53.9% for Medicaid [35].

Our project assumed a "base" scenario in which screening rates using the HEDIS definition for white women were 80% and African-American women were 70%. Reducing the disparity in screening rates would involve raising the rate of screening in African-American women from 70% to 80%. The consequences of mammography rates of 60% and 90% were also examined to expand the range of applicability of the model.

The HEDIS measure definition only requires a mammogram once in two years rather than annually. Because most national guidelines recommend annual mammograms for women in the 50-65 age group, and because the structure of the Markov model was built around annual cycles, it was necessary to identify patterns of annual, biennial, and less frequent mammograms that would be consistent with published literature on mammography screening and on the observed HEDIS mammography rates.

These patterns were estimated using data from Blanchard et al [63], who reported that twice as many African-American women who had had at least one mammogram in five years had biennial vs. annual mammograms. Among women having any mammograms in five years, the proportions with annual, biennial, or "sporadic" mammograms (defined as one in five years) were .23, .46, and .31, respectively. These ratios were used to estimate the proportion of women in a health plan population who would have to receive annual, biennial, "sporadic," or no mammograms to obtain HEDIS two-year mammography rates of 60%, 70%, 80%, or 90% [Additional File 1: Appendix Table 1].

For sake of simplicity, it was assumed that women's screening patterns would remain the same over the five-year model period. In subsequent model cycles, then, the probability of mammography was dependent on the screening pattern assigned in the initial cycle and whether or not a mammogram had occurred in the most recent cycle.

In one of the models run for sensitivity analysis, the probability of mammography in a later cycle after a false positive was increased by 10% in relative terms (e.g., from .182 to .20) [50].

##### Cancer Incidence

Mammography screening detects either newly incident cancers, cancers missed in earlier screening rounds, or prevalent cancers in women who have not had previous mammograms. In a population of women who are members of an HMO and who are generally getting regular screening, most of the cancers identified in any given year will be incident cases. Among women having annual mammograms, then, the number of cancers identified in any cycle can be estimated by using data on annual incidence of cancers among African-American women in the relevant age bands. Incidence rates can also be estimated by published studies of cancers identified in mammography screening programs.

The annual incidence rate for African-American women aged 50-65 represents a lower bound on an estimate of number of cancers identified through mammography, since it would presume no "old" cancers to be found. SEER data from the National Cancer Institute provide several estimates of annual incidence for African-American women in the 55-59 or 55-60 age band (representing the middle of the HEDIS age group): .00267 [64], .00275 [65], .00282 [66]. The minor variation seems to reflect the specific time period of SEER data being examined. Leung et al [67] also provide an estimate of annual incidence for African-American women aged 50-69 as .0025.

There are several published studies of cancers detected during mammography screening programs for African-American women. Estimates range from 1.12 to 21 cancers per 1,000 women screened. The lowest estimate is for interval cancers between mammograms two years apart; the highest estimate is for first screens among women with a family history of cancer and generally no previous mammograms. Two of the studies found 3 cancers per 1,000 mammograms in second screening rounds [68,69]; this situation is perhaps most closely analogous to the HMO scenario being modeled in which most women will have had one or more previous mammograms.

The base case scenario used 3 cancers per 1,000 as the estimate of annual incidence in a population of African-American HMO members aged 50-64 who receive either annual or biennial mammograms. Five cancers per 1,000 mammograms was the incidence estimate for women with sporadic mammography patterns; 8 cancers per 1,000 for women having cancer detected clinically in an annual cycle in which they did not receive a mammogram. The rate of 8 per 1,000 rate was chosen as a middle-range estimate between 5 per 1,000 and the 10 per 1,000 rate reported by Kerlikowske et al [69] for first screenings.

##### Stage at Diagnosis with Mammography

Several studies [70-74] report distributions of stage of cancer at diagnosis for African-American women receiving mammograms [Additional File 1: Appendix Table 2].

Data from Jacobellis and Cutter [70] [Additional File 1: Appendix Table 2] represent a relatively favorable estimate of distribution of stage at diagnosis in stages III and IV, but have a relatively low percentage of cases identified as cancer *in situ*. These figures were chosen for the base case model for women with annual or biennial mammograms because they represented a middle-range estimate of the benefit of mammography. We used the same stage distribution for the annual and biennial schedules because of the finding of White et al [75]. The stage distribution reported by Yood et al [72] was used in the base case model for the "sporadic" mammogram scenario, since it was derived from a population of African-American women who were members of a managed care plan, with a range of mammography screening patterns. Data from Bibb [74] in Table 2 represent a "best case" estimate of stage distribution; these figures were used in sensitivity analyses as an upper bound of the benefit of mammography for improving stage at diagnosis (see later section on sensitivity analysis).

##### Stage at Diagnosis without Mammography

Fewer studies are available with stage distributions for African-American women diagnosed without mammography. Some of the studies combine *in situ *and Stage I diagnoses as "early stage" and Stages II-IV as "advanced stage." One of these studies found 36% of women with early stage disease and 64% with advanced stage disease [76]. One study with a detailed stage distribution [74] found the following distribution of stage at diagnosis: *In Situ - *4%; Stage I - 32%; Stage II - 43%; Stage III - 13%; Stage IV - 8%. This distribution is closely matched to the Moorman et al [74] distribution of "early" vs. "late," so we used this second Bibb [74] stage distribution as the estimate for women not receiving mammography.

##### Remission, Progressive Disease, or Recurrence, and Deaths Due to Cancer

Breast cancer can follow a complex course of response to treatment, recurrence, remission, progression, and eventual death. To simplify the potential sequence of events, the Markov model presumed that women with newly diagnosed cancer will be treated, and the treatment will produce either a remission or progressive disease. In subsequent cycles, women in remission are presumed to either stay in remission or have recurrent illness. The probability of staying in remission varied in the model as a function of stage at initial diagnosis - remission continued for 98.5% of women with Stage I disease but only continued for 67% of women with Stage IV disease. Remission either continued or shifted to recurrent disease at each year of the five-year model. Women who had a recurrence in any one of the five years may have died in that year or may have continued treatment with some probability of a second remission.

Probabilities for all specific remission, treatment, or recurrence branches were selected to match as closely as possible the published five- and six-year survival rates for African-American women with breast cancer, by stage at diagnosis [73,77]. Annual death rates in the model, by stage, were: Stage I - 0.75%; Stage II - 3%; Stage III - 12.6%; Stage IV - 26.4%. To produce these rates in years 2-5 of the model when women diagnosed in previous years and having an initial remission can experience recurrences and subsequent progressive disease, the probabilities of continued remission in each subsequent year, by stage, were: Stage I - 97.5%; Stage II - 94%; Stage III - 81%; Stage IV - 66%.

##### Deaths from Other Causes

In all years of the model, a probability of .01 was assigned to risk of death from causes other than cancer [78,79].

#### Values for Health States

##### Mortality/Survival

For mortality/survival analyses, all states other than death were assigned a value of 1; death was assigned a value of 0. Values were assigned at each annual cycle of the model. Over five years, then, the model calculates life expectancy on a scale from 0-5. If all women died in beginning of the first year of the model, the total life expectancy would be 0; if all women survived for five years (even if many were being treated for active cancer), the total life expectancy would be 5.0.

##### Utilities

For analysis of Quality-Adjusted Life Years, utilities were assigned to each health state in which a woman could start or end a year [Additional File 1: Appendix Table 3] [80-86]. Different utility values were assigned to women in remission from different stage of cancer to reflect negative health impacts of breast reconstruction surgery, adjuvant chemotherapy, long-term effects of intensive chemotherapy, and other health effects associated with treatment of more advanced disease.

The simulation model software (TreeAge Pro, TreeAge Software) [87] allows for "transition states" that can either add or subtract utility values during part of a year even if individuals in the model do not end a model cycle and start a new model cycle in that state. In our model, "active treatment" was modeled in this way; subtractions of .10, .20, and .25 were made from the utility values for women who started the year in the well state and had treatment for Stage I, Stage II, or Stages III or IV cancer during that year.

##### Probability of Employment

There were a relatively small number of studies describing labor force participation among minority women with breast cancer. Not all of these presented data separately by stage of disease or presence of active treatment, so estimates for the model had to be made by combining parameters from different studies. Our model presumed a cohort of women who were all working full-time at the start of the simulation to estimate a business case for eliminating disparities in mammography from the employer, rather than societal, perspective.

Bradley et al [88,89] reported a reduction in likelihood of employment for women with cancer in the past two years compared to women without cancer in the same time period (54% vs. 64%). The difference was 6% in women who had cancer three or more years prior to the study date. Short et al [90] reported a similar 8% decrease in the probability of working as a function of having cancer; in addition, the relative probability of working decreased as a function of stage at diagnosis. More recently, Bradley et al [91] reported that among women with breast cancer employed prior to diagnosis, at least 12% appeared to move out of the labor force altogether by retiring or becoming disabled. The effects were stronger for those with more advanced stage cancers, while there was no employment effect on women with in situ cancers. The non-employment effects of breast cancer were about twice as strong for African-American women.

Eversley [92] reported that African-American women took eight more weeks off after surgery than white women and that 44% of all women were not working at three months after diagnosis. Satariano and DeLorenze [93] reported that 60% of black women vs. 74% of white women were back at work three months after diagnosis; return to work also varied as a function of stage of disease at diagnosis, with 76% of women with "local" disease returning to work at three months, while 69% of women with "regional" disease and 50% of women with "remote" disease were back at work.

Only a few studies have examined absenteeism among breast cancer survivors. Bradley et al [94] found that women treated for breast cancer missed an average of 44.5 days from work and women with late stage disease missed far more days from work than women with in situ cancer.

We combined work loss hours estimates (Bradley et al) with annual earnings (or wages) data (Bureau of Labor Statistics) to calculate the monetary value of productivity losses due to absenteeism for survivors who are employed and in treatment.

### II. Asthma Medications Model

#### Rates of Use of Appropriate Medications

Several published studies have shown racial/ethnic disparities in the use of IHC or other preventive medications (e.g., cromolyn) for asthma [36,37,95]. The relationship between some of these findings and the HEDIS measure of appropriate asthma medications is complicated because the HEDIS measure only requires a single prescription to be filled during a year, and some of the studies use regular ICS use as the key measure of medication use. Rates of regular ICS use will inevitably be lower than rates of having received at least one prescription for preventive medications.

The NCQA State of Health Care report for 2008 shows a median rate of appropriate medication use according to the HEDIS measure definition of approximately 92% across all three age groups for commercial enrollees and 87% for Medicaid [35]. There is considerable variability in these rates from plan to plan [96]. These rates are for all plan members and are not stratified by race/ethnicity.

Studies comparing medication use across racial/ethnic groups report odds ratios of appropriate medication use between .36 and .65 when comparing African-American adults to a non-Hispanic white reference group [97,98]. The study by Ortega et al [36] found medication use rates for African-American children and Hispanic children at approximately half of the rate for non-Hispanic white children; smaller disparities were reported for children and adolescents in a Medicaid sample by Lieu et al [95]. A more recent study by Smith et al [99] found a 12% absolute disparity between black and white children in a measure of underuse of controlled medications.

The baseline scenario for the asthma model, then, starts with a rate of appropriate medication use for minority adults and children of 80% and posits a rate of 90% for non-Hispanic white children and adults. Elimination of the disparity involves raising the rate of medication use in minority adults and children to 90%.

#### Acute Exacerbations

Although there can be some asthma-related symptoms and related lung function deficits on "normal" days, most problems of lost work or school or need for medical care come as a result of acute exacerbations caused by some environmental trigger. Some exacerbations are severe enough to require an ER visit or hospital admission.

Monthly probabilities of exacerbations leading to at least one day off work or school were estimated from studies using patient surveys to inquire about either days off work or school or "physically unhealthy days" in a given time period. In the three most directly relevant studies, the monthly probabilities of at least one day of work or school missed due to acute exacerbations were .30 [95], .50 [98], and .37 [32]. We chose to use a probability in the middle of this range: .40. These studies included patients with all levels of medication use. To be consistent with the Sin et al report of a 50% decrease in acute exacerbations through use of inhaled corticosteroids [100,101], we used a monthly probability of acute exacerbations of .27 for those taking medications and .53 for those not taking medications.

Five recent studies included data on monthly probabilities of ER or urgent care visits, with a range of probabilities from .01 to .06 [49,95,98,102-104]. These studies include patients of all severity levels and degrees of compliance with medications. The ER visit rates for patients with persistent asthma only (HEDIS denominator population) would be slightly higher. The probability of an ER visit is higher for African-American or Hispanic children and adults, with one study in a managed care setting showing ER visit rates twice as high in African-American patients as in non-Hispanic white patients.

A recent review of clinical trials of inhaled corticosteroids and other preventive medications suggested that use of these medications cut the rate of acute exacerbations in half [101]. It is not clear exactly how to model the effect of just barely meeting the HEDIS requirement of one filled prescription in a year, but our "base case" model presumes that most patients with asthma in a HEDIS denominator population who receive a prescription are taking medications as regularly as patients in the studies reviewed. In sensitivity analyses, we model the effects of a smaller benefit related to poorer levels of adherence.

To maximize comparability of our results to those of Paltiel et al [49], we chose to use rates from their model, combining the probability of ER and urgent care use. We multiplied those rates by two to estimate the probability of ER/urgent care visits for African-American or Hispanic adults and children. The monthly probabilities in the base model are .038 for patients using medications and .076 for patients not using medications.

Monthly probabilities of hospital admission were also derived from rates used by Paltiel et al [49], with adjustment for the finding in several published studies of higher hospitalization rates for African-American or Hispanic patients with asthma. Monthly probabilities in those studies ranged from .003 to .010. The ratio of rates for patients using vs. not using medications were also adjusted from those used by Paltiel et al [49] to match the recent summary by Sin et al [101] showing a decrease in hospitalization rates of approximately 50% in patients using inhaled corticosteroids. Our base model monthly probabilities for hospital admission were .0035 for patients using medications and .0069 for patients not using medications.

#### Days Missed from Work or School

For adults, we used 21.7 as the estimate of the number of days per month potentially available for attendance at work (2080 hours as a full-time work year = 260 days per year = 21.67 days per month). For children and adolescents, we used 15 as the estimate of the number of days per month potentially available for attendance at school (180 school days in a year = 15 days per month). The 180 school days are actually not evenly distributed across all 12 months of the year, so the effects of exacerbations are clearly more important on lost school days between September and June than they are in the summer. However, we did not have any data that would allow us to model seasonal variation in rates of exacerbation by race/ethnicity, so for purposes of creating a base model we chose to model days off school as if those days were distributed over an entire year. The model will therefore underestimate the effect of exacerbations on lost school days for 9-10 months of the year and overestimate the effect for 2-3 months, but the average impact over the entire year should be reasonably accurate.

The base model presumes that an average of four days of work or school are lost per month among those patients having acute exacerbations [32,95,98]. It presumes an additional day per month lost for those patients requiring an ER or urgent care visit for an exacerbation. It presumes an additional three days lost for those patients requiring hospital admission for an exacerbation. The number of days lost is not presumed to vary for members of different racial/ethnic groups. There are studies showing more "physically unhealthy days" or days lost from work or school for African-American or Hispanic patients with asthma [6], but we are presuming that this is due to the higher probability of exacerbations rather than a greater number of days lost from work or school given an exacerbation.

#### QALYs

We used utility values from the Paltiel et al [49] model to estimate the decrement in health status produced by acute exacerbations, ER visits, and hospitalizations. We also used their estimate of a .01 decrement in utility related to the use of medications. The utility value assigned to a "normal" day without medications in the model is .92; the value is .91 for a "normal" day with medications. The utility value for a day with an exacerbation leading to being off from work or school is .72. (We did not subtract the .01 for medication use on these days, on the argument that the "disutility" of an acute exacerbation would dominate any minor decrement in health status due to side effects of medications on those days.) We subtracted an additional .05 on days in which an exacerbation required an ER visit, and an additional .10 for days hospitalized. Utilities on ER days, then, were .67 and on hospitalized days, .62. In sensitivity analyses, the disutility of an ER visit or hospitalization was increased to .10 and .20, respectively. The utility values for a month in various branches of the model were derived by multiplying the utilities for each type of day by the number of days per month estimated for each of those states (see previous section).

For patients in the model who died in a given month, a utility of .92 or .91 (depending on medication use) was assigned for 15 days and 0 for the remainder.

#### Deaths

We presumed that asthma-related deaths would only occur among those patients experiencing exacerbations requiring an ER visit or a hospital admission. The probability of deaths in these groups was estimated at .0002 for patients requiring ER visits and .0014 for patients requiring hospitalization. Given the relatively low probability of ER visits or admissions, the model's estimates for mortality in the entire denominator population are reasonably consistent with those of Suissa et al [29] and Paltiel et al [49]. The probability of death from causes other than asthma was estimated at .0001 per month.

#### Transition Probabilities

Patients are not consistent from month to month or year to year in their use of medications. Although a rate of adherence may be stable over time for a large population, individuals can alternate between periods of use and non-use, and the Markov model structure allows for this factor to be included. We presumed that medication use would be generally stable from month to month, but that some individuals taking medication and experiencing no exacerbations in a given month would not use medications in the following month (in various versions of the model we used proportions of 5-10%). Even some individuals experiencing ER visits or hospitalizations may not use medications in the following month (perhaps believing them to be ineffective); we estimated this proportion as 1%, with 99% of those experiencing ER visits or hospitalizations continuing on medications.

Among those not using medications in a given month, we presumed that 1% of those not experiencing exacerbations would begin to use medications in the following month, that 6% of those experiencing an exacerbation would begin to use medications in the following month, and that 25% of those requiring an ER visit or hospitalization would begin using medications in the following month.

In the absence of published data on these transitions among minority group patients with asthma, some of the transition probabilities are somewhat arbitrary. They were adjusted, though, to produce the effect of gradually rising use of medication for the group as a whole over time. This trend of gradual increase is consistent with national data on improvement in the HEDIS asthma medication measure over time [96].

## Pre-publication history

The pre-publication history for this paper can be accessed here:

http://www.biomedcentral.com/1471-2288/11/31/prepub

## Supplementary Material

Additional file 1 **Appendix Tables**. Three tables that accompany the Appendix text. Click here for file
