# Supplementary material for: A simulation model approach to analysis of the business case for eliminating health care disparities
Source: BMC Med Res Methodol. 2011 Mar 19;11:31. doi: 10.1186/1471-2288-11-31 (PMC3073955; doi:10.1186/1471-2288-11-31)
Supplement: Additional file 1 — Appendix Tables. Three tables that accompany the Appendix text. [file 1471-2288-11-31-S1.DOC]

Appendix Table 1. Probabilities of annual, biennial, and sporadic mammograms included in model to match HEDIS Mammography rates of 60-90%

|  | **HEDIS Mammography Rate** | | | |
| --- | --- | --- | --- | --- |
| **Initial Model State** | **60%** | **70%** | **80%** | **90%** |
| **Annual Mammogram** | .152 | .197 | .242 | .288 |
| **Biennial Mammogram** | .304 | .395 | .483 | .573 |
| **“Sporadic” Mammogram** | .360 | .270 | .182 | .092 |
| **No Mammogram** | .184 | .138 | .093 | .047 |

Appendix Table 2. Estimates from published sources on stage distribution of newly diagnosed breast cancer in African-American women.

| **Stage** | **Jacobellis & Cutter [70]** | **Jacobellis [70] (incident cases)** | **May et al. [68]** | **Li et al. [71]** | **Yood et al. [72]** | **Chu et al. [73]** | **Bibb [74]** |
| --- | --- | --- | --- | --- | --- | --- | --- |
| In Situ | 16 | 13 | 24.8 | - | 17 | - | 21 |
| I | 35.4 | 41.3 | 24.2 | 35.4 | 29 | 37.2 | 57 |
| II | 38.9 | 37 | 29.7 | 45.7 | 40 | 44.6 | 16 |
| III | 6.9 | 6.5 | 11.7 | 11 | 9 | 10.2 | 4 |
| IV | 2.8 | 2.2 | 7.2 | 7.9 | 5 | 8.0 | 2 |

Appendix Table 3. Utility values assigned to health states in mammography simulation model

| **Health State** | **Utility Assigned** | **Range in Published Literature [80-86]** |
| --- | --- | --- |
| Well | 1.0 | - |
| In Remission – Stage I | .90 | .80 - .98 |
| In Remission – Stage II | .85 | .80 - .98 |
| In Remission – Stage III | .81 | .80 - .98 |
| In Remission – Stage IV | .81 | .80 - .98 |
| Progressive Disease | .40 | .30 - .69 |
| Death | 0 | - |
